# Dynamic changes in peripheral blood lymphocyte subsets predict the efficacy and prognosis of immune checkpoint inhibitors in metastatic osteosarcoma

**DOI:** 10.3389/fimmu.2026.1766639

**Published:** 2026-05-13

**Authors:** Longqing Li, Jinlei Liu, Yuan Zhao, Songtao Pang, Bin Zhang, Jia Wen, Yongkui Liu, Yi Zhang, Yan Zhang, Jiazhen Li, Nan Zhou, Xinchang Lu

**Affiliations:** Department of Orthopedics, The First Affiliated Hospital of Zhengzhou University, Zhengzhou, China

**Keywords:** biomarkers, immune checkpoint inhibitors, metastatic, osteosarcoma, peripheral blood lymphocyte subsets

## Abstract

**Background:**

Osteosarcoma (OSA) remains the most common primary malignant bone tumor in children and adolescents, with a poor prognosis for metastatic disease. While immune checkpoint inhibitors (ICIs) have revolutionized cancer treatment, their efficacy in metastatic OSA is limited and highly variable, lacking clear predictive biomarkers. Peripheral blood lymphocyte subsets, reflecting systemic immune status, show promise as non-invasive predictors of ICIs efficacy and patient prognosis, yet their dynamic changes and predictive value in metastatic OSA are largely unexplored.

**Methods:**

This single-center, retrospective study included 14 metastatic OSA patients receiving ICIs. Peripheral blood lymphocyte subsets (CD3^+^, CD4^+^, CD8^+^ T cells, NK cells, and activated T cells like CD4^+^HLA-DR^+^, CD8^+^HLA-DR^+^) were quantified by flow cytometry at diagnosis, pre-ICIs, and post-ICIs. Overall survival (OS) and treatment response (RECIST 1.1) were evaluated. Statistical analyses, including univariate Cox regression, group comparisons, and Linear Mixed Models (LMM), assessed the relationship between lymphocyte subsets, their dynamic changes, and clinical outcomes.

**Results:**

Univariate Cox regression identified pre-ICIs CD8^+^HLA-DR^+^ cells, and post-ICIs CD3^−^CD56^+^, CD3^+^CD4^+^, and CD8^+^HLA-DR^+^ cells as protective factors for OS. At diagnosis, CD45^+^, CD3^−^CD56^+^, CD4^+^HLA-DR^+^, CD3^+^CD8^+^, and CD8^+^HLA-DR^+^ cells were significantly higher in patients achieving disease control rate (DCR) versus progressive disease (PD). Post-ICIs, CD3^−^CD56^+^, CD3^+^CD4^+^, and CD8^+^HLA-DR^+^ cells were significantly elevated in the DCR group. LMM revealed dynamic increases in post-ICIs CD3^−^CD56^+^ and CD8^+^HLA-DR^+^ cells were significantly associated with better treatment response (Time Point × Response P < 0.001 and P = 0.003). Relative increases in post-ICIs CD3^−^CD56^+^, CD3^+^CD4^+^, and CD8^+^HLA-DR^+^ cells were protective for OS. Extrapulmonary metastasis was a significant risk factor for poor OS (HR: 6.75, P = 0.040).

**Conclusion:**

This study provides the first systematic analysis of dynamic changes in peripheral blood lymphocyte subsets in metastatic OSA patients undergoing ICIs. Our findings suggest that elevated proportions and dynamic increases of peripheral blood CD3^−^CD56^+^ (NK cells) and CD8^+^HLA-DR^+^ (activated cytotoxic T cells) after ICIs are consistently associated with improved OS and better treatment response. These accessible biomarkers hold potential for predicting ICIs efficacy and guiding individualized treatment strategies in metastatic OSA, warranting further validation in larger, prospective cohorts.

## 1 Introduction

Osteosarcoma (OSA) is the most common primary malignant bone tumor, predominantly affecting children and adolescents (1, 2). Despite multimodal therapy, including neoadjuvant chemotherapy, surgery, and adjuvant chemotherapy, which has improved the 5-year overall survival (OS) rate for patients with non-metastatic OS to over 60-70%, the prognosis remains extremely poor for patients presenting with distant metastasis at diagnosis or developing metastasis after treatment, with 5-year OS rates below 20% (3, 4). This stark contrast highlights the significant challenges in treating metastatic OS and underscores the urgent need for novel therapeutic strategies.

In recent years, immune checkpoint inhibitors (ICIs) have demonstrated breakthrough efficacy in various common solid tumors by activating the host anti-tumor immune response through the blockade of pathways such as PD-1/PD-L1 or CTLA-4 (5, 6). However, the application of ICIs in rare tumors, particularly in the field of sarcomas including OSA, remains in the exploratory stage. Clinical trial data indicate significant heterogeneity in the efficacy of ICIs across different sarcoma subtypes: for instance, ICIs have exhibited an objective response rate (ORR) exceeding 50% in alveolar soft part sarcoma (7, 8); in contrast, unselected OSA cohorts show extremely limited response to ICI monotherapy, with only a small minority of patients achieving clinical benefit (9–16). Although combination therapeutic strategies have improved efficacy to some extent, the overall ORR remains at a low level (10). The current status clearly indicates an urgent need to identify precise predictive biomarkers for OSA immunotherapy to screen for populations that may potentially benefit (17).

Nowadays, the development of predictive biomarkers for OSA patients has become critical for achieving precision therapy with ICIs. However, established biomarkers widely utilized in common malignancies, such as PD-L1 expression and tumor mutational burden (TMB), exhibit limited predictive efficacy in OSA. Although recent studies have proposed a range of multigene signature markers and demonstrated encouraging predictive potential in sarcoma patient cohorts, their clinical application remains severely hampered by difficulties in obtaining OSA tissue samples and the lack of standardized detection platforms (18, 19). Given that systemic immune status is closely correlated with the composition and functional status of immune cells within the tumor microenvironment (TME) (20, 21), and that the latter is a central determinant of ICIs efficacy, immune monitoring via peripheral blood represents an ideal entry point for developing predictive biomarkers (22, 23). Peripheral blood lymphocyte subset analysis, as a non-invasive and convenient diagnostic approach, has been confirmed to correlate closely with ICIs efficacy in various tumors (24–26), however, its dynamic evolution and prognostic value in patients with metastatic OSA have not yet been fully elucidated.

Based on the aforementioned background, this study aims to systematically investigate the dynamic changes in peripheral blood lymphocyte subsets in patients with metastatic OSA undergoing ICIs therapy. By analyzing the longitudinal profiles of these immune cells at diagnosis, pre-ICIs, and post-ICIs, we seek to identify potential peripheral blood biomarkers associated with treatment response and prognosis. This study serves as a preliminary exploratory analysis, providing initial evidence on the immunological landscape of metastatic OS patients treated with ICIs. Our findings may offer insights into the role of systemic immune monitoring and lay the groundwork for future larger-scale, prospective studies to validate these biomarkers for clinical decision-making.

## 2 Patients and methods

### 2.1 Study design and patient selection

This single-center, retrospective study aimed to evaluate the efficacy of ICIs in patients with metastatic OSA and to assess the predictive value of peripheral blood lymphocyte subsets. We retrospectively collected clinical data from patients with metastatic OSA who received ICIs therapy at the Bone and Soft Tissue Tumor Center of the First Affiliated Hospital of Zhengzhou University between October 2023 and April 2025. Patients were included based on the following criteria: 1) Histopathologically confirmed high-grade OSA and diagnosed with metastatic disease during the study period. 2) The patient agrees and received at least one cycle of immune checkpoint inhibitor therapy (200 mg camrelizumab intravenously, every 2 weeks, with a cycle of 4 weeks). 3) Had complete peripheral blood lymphocyte subset data available at diagnosis, before the first immunotherapy, and after the first immunotherapy. 4) Had complete clinical follow-up data, including treatment response and survival outcomes. The exclusion criteria for patients were as follows: 1) Patients with low-grade OSA or localized high-grade OSA. 2) Patients who did not receive standard ICIs therapy or received non-standard ICIs treatment regimens. 3) Patients with other hematological diseases or autoimmune diseases that may affect immune status. 4) Patients with other concurrent malignancies.

We defined the three time points as follows: At Diagnosis: Blood samples were collected at the time of confirmed metastatic osteosarcoma diagnosis. Pre-ICIs: Samples were collected within 7 days prior to the first dose of ICIs therapy, as part of the routine clinical assessment (including blood counts, liver/renal function, and CT scans) to establish a baseline for immunotherapy. Post-ICIs: Samples were collected after the completion of the first cycle of ICIs (typically 3days post-treatment).

In total, 14 patients met all inclusion criteria and none of the exclusion criteria, and were thus included in this study. All enrolled patients were followed up regularly until death or October 2025, whichever occurred first. This study was approved by the Ethics Committee of the First Affiliated Hospital of Zhengzhou University.

### 2.2 Clinical data collection

We collected detailed clinicopathological characteristics for all included patients, including age, gender, presence of extra-pulmonary metastases, prior treatment history (e.g., second-line chemotherapy, anlotinib use). The primary endpoint was OS, defined as the time from the start of the first ICIs treatment to death from any cause. The secondary endpoint was the treatment response.

### 2.3 Evaluation of treatment response

Treatment responses were evaluated according to the Response Evaluation Criteria in Solid Tumors (RECIST) version 1.1. Responses were classified as complete response (CR), partial response (PR), stable disease (SD), and progressive disease (PD). In this study, the disease control rate (DCR) was defined as the proportion of patients who achieved CR, PR, or SD. Progressive disease (PD) was defined as an increase in tumor burden or the appearance of new lesions.

### 2.4 Flow cytometry analysis of peripheral blood lymphocyte subsets

Peripheral blood lymphocyte subset analysis was performed as follows. First, blood samples were collected from patients via peripheral venipuncture at diagnosis, before the first ICIs treatment, and after the first ICIs treatment. Subsequently, red blood cells were lysed from whole blood using red blood cell lysis buffer, resulting in a white blood cell suspension. The white blood cell suspension was then stained with a panel of fluorescently labeled monoclonal antibodies, following the manufacturer’s instructions, to specifically identify different lymphocyte surface antigens. Stained samples were analyzed using a BD FACS Canto II flow cytometer for detection and data acquisition. Finally, the data were analyzed using specialized software to obtain and record the relative percentages of key lymphocyte subsets, including: CD45^+^ leukocytes (used for initial gating), CD3^+^ T cells, CD3^+^CD4^+^ helper T cells (CD4^+^ T cells), CD3^+^CD8^+^ cytotoxic T cells (CD8^+^ T cells), CD3^-^CD56^+^ Natural killer cells (NK cells), as well as subsets indicative of T-cell activation and functional status, including CD4^+^CD28^+^, CD8^+^CD28^+^, CD4^+^CD38^+^, CD8^+^CD38^+^, CD4^+^HLA-DR^+^, and CD8^+^HLA-DR^+^ cells. The percentages of cell subsets were calculated based on their proportion within the lymphocyte-gated region.

### 2.5 Statistical analysis

All statistical analyses and visualizations in this study were performed using R software (4.4.2). The normality of continuous variables was assessed using the Shapiro-Wilk test. Normally distributed data are presented as mean ± standard deviation, while non-normally distributed data are presented as median (interquartile range). Group comparisons were performed using appropriate statistical tests based on the data distribution characteristics: independent samples t-tests for normally distributed data and Mann-Whitney U tests for non-normally distributed data. Categorical data are presented as frequencies (percentages), and group comparisons were performed using chi-square tests or Fisher’s exact tests. OS curves were plotted using the Kaplan-Meier method. Cox proportional hazards regression models were used to identify prognostic factors.

To assess the trends of lymphocyte subsets over time in different treatment response groups (DCR group vs. PD group), a Linear Mixed Model (LMM) was employed. The LMM effectively handles repeated measures data and accounts for inter-individual and intra-individual variability, allowing for a more accurate analysis of the dynamic changes in lymphocyte subsets at different time points (before and after ICIs). The model included time point, treatment response group, and the interaction between the two as fixed effects, and patient ID as a random effect. The absolute changes and relative percentage change for each lymphocyte subset after ICIs treatment compared to before ICIs treatment was also calculated. The Absolute changes = (Post-ICIs minus Pre-ICIs). The relative change was calculated as follows: Relative change = (value after ICIs treatment - value before ICIs treatment)/value before ICIs treatment × 100%. All analyses were two-sided, and P values < 0.05 were considered statistically significant.

## 3 Results

### 3.1 Patient baseline characteristics

This study enrolled a total of 14 patients with metastatic OSA who received ICIs therapy. The clinical characteristics of the patients are presented in **Table 1**. The median age of the patients was 15 years (range: 9–22 years), with 9 males (64.3%) and 5 females (35.7%). Ten patients had lung-only metastases, while 4 patients presented with extra-pulmonary metastases. Regarding prior treatments, 3 patients had received second-line chemotherapy, and 5 patients had previously used anlotinib. All patients received ICIs therapy.

**Table 1:** Baseline clinicopathological characteristics of metastatic OSA patients (N = 14).

Characteristic	N (%) or median (range)
Demographics	
Age (years)	15 (9-22)
Gender	
Male	9 (64.3%)
Female	5 (35.7%)
Disease Characteristics	
Metastasis Site	
Lung only	10 (71.4%)
Extrapulmonary	4 (28.6%)
Prior treatment history	
Second-line chemotherapy	3 (21.4%)
Anlotinib use	5 (35.7%)
Follow-up and outcomes	
Median Follow-up (months)	14 (6-24)
Survival Status at Cut-off	
Alive	5 (35.7%)
Deceased	9 (64.3%)

**Figure 1:**
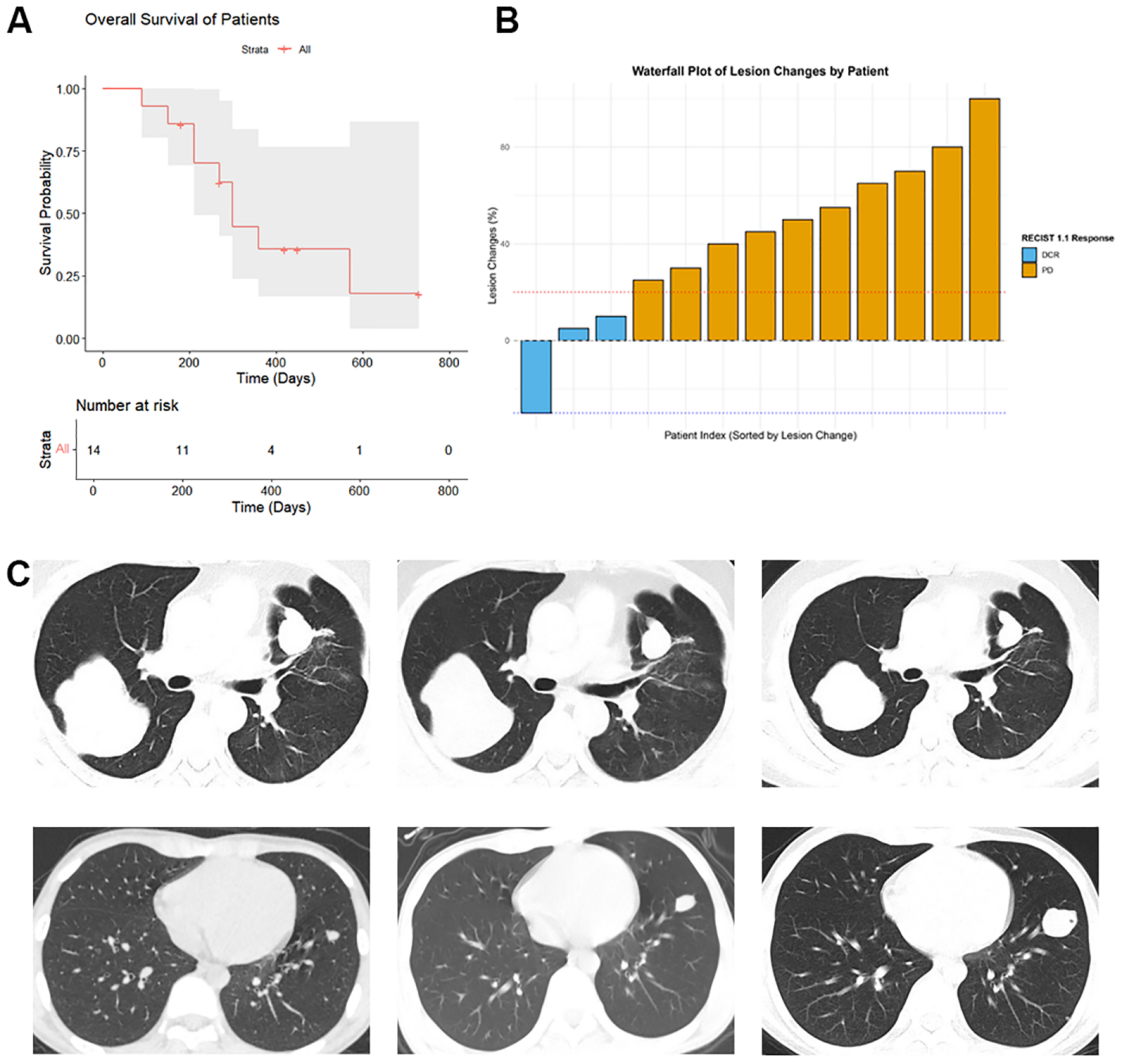
Overall survival, treatment response, and representative CT images of metastatic OSA patients receiving ICIs. **(A)** Overall survival curve of 14 metastatic OSA patients treated with ICIs. The Kaplan-Meier curve illustrates the probability of survival over time for the entire cohort; **(B)** Waterfall plot depicting the best treatment response of 14 metastatic OSA patients to ICIs therapy. Tumor response was evaluated according to RECIST 1.1 criteria. Each bar represents an individual patient, with the length and direction indicating the percentage change in tumor size from baseline. Positive values indicate tumor progression, while negative values indicate tumor shrinkage; **(C)** Representative computed tomography images of lung metastases before and after ICIs treatment. Upper panel: CT images from a DCR patient. Lower panel: CT images from a PD patient.

At the time of follow-up cutoff, the median follow-up duration was 14 months (range: 6–24 months). Among these, 5 patients were alive, and 9 patients had died (**Figure 1A**). According to RECIST 1.1 criteria, 3 patients achieved DCR. Unfortunately, no CR cases were observed; specifically, 1 patient achieved a PR, and 2 patients exhibited SD. The remaining 11 patients experienced PD (**Figure 1B**). **Figure 1C** illustrates the pre- and post-ICIs treatment lung CT scan results for one DCR patient and one PD patient. Due to the limited sample size of this study (N = 14), the findings presented should be considered preliminary and hypothesis-generating.

### 3.2 Relationship between lymphocyte subsets and overall survival

**Figure 2:**
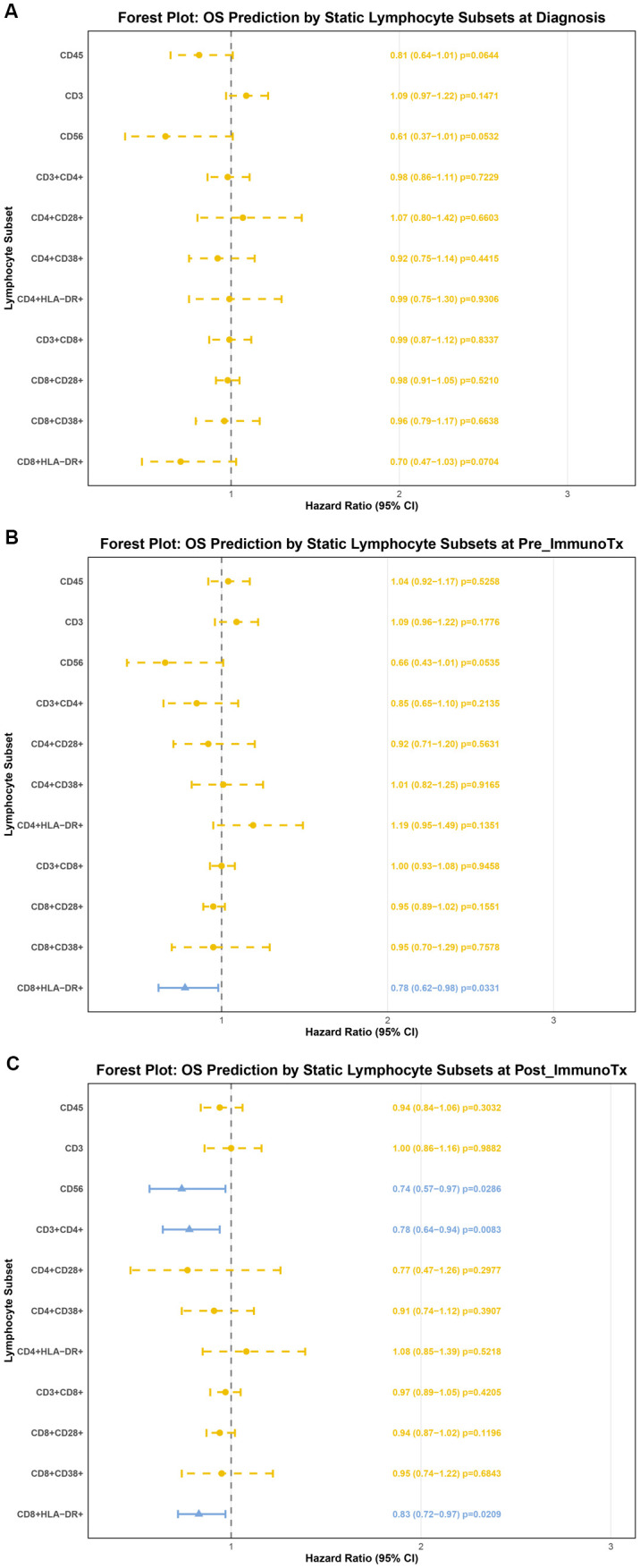
Forest plots illustrating the association between peripheral blood lymphocyte subsets and OS in metastatic OSA patients. **(A)** Forest plot showing the univariate Cox regression analysis of lymphocyte subset proportions at diagnosis and patient OS; **(B)** Forest plot showing the univariate Cox regression analysis of lymphocyte subset proportions before ICIs treatment and patient OS; **(C)** Forest plot showing the univariate Cox regression analysis of lymphocyte subset proportions after ICIs treatment and patient OS. Each horizontal line represents a lymphocyte subset, with the square indicating the HR and the whiskers representing the 95% CI. Variables colored in yellow indicate a P-value > 0.05, while variables colored in blue indicate a P-value < 0.05, suggesting a statistically significant association with OS.

We analyzed peripheral blood lymphocyte subset levels in patients at three time points: diagnosis, before ICIs treatment, and after ICIs treatment. Through univariate Cox regression analysis, we found no significant relationship between lymphocyte subset proportions at diagnosis and patient OS. Before immunotherapy, CD8^+^HLA-DR^+^ cells were identified as a protective factor for metastatic OSA patients undergoing ICIs treatment (HR: 0.78 (0.62-0.98), p=0.033). After ICIs treatment, CD3^-^CD56^+^ cells (HR: 0.74 (0.57-0.97), p=0.028), CD3^+^CD4^+^ cells (HR: 0.78 (0.64-0.94), p=0.008), and CD8^+^HLA-DR^+^ cells (HR: 0.83 (0.72-0.97), p=0.021) were protective factors for patients (**Figures 2A–C**).

### 3.3 Relationship between lymphocyte subsets and treatment response

**Figure 3:**
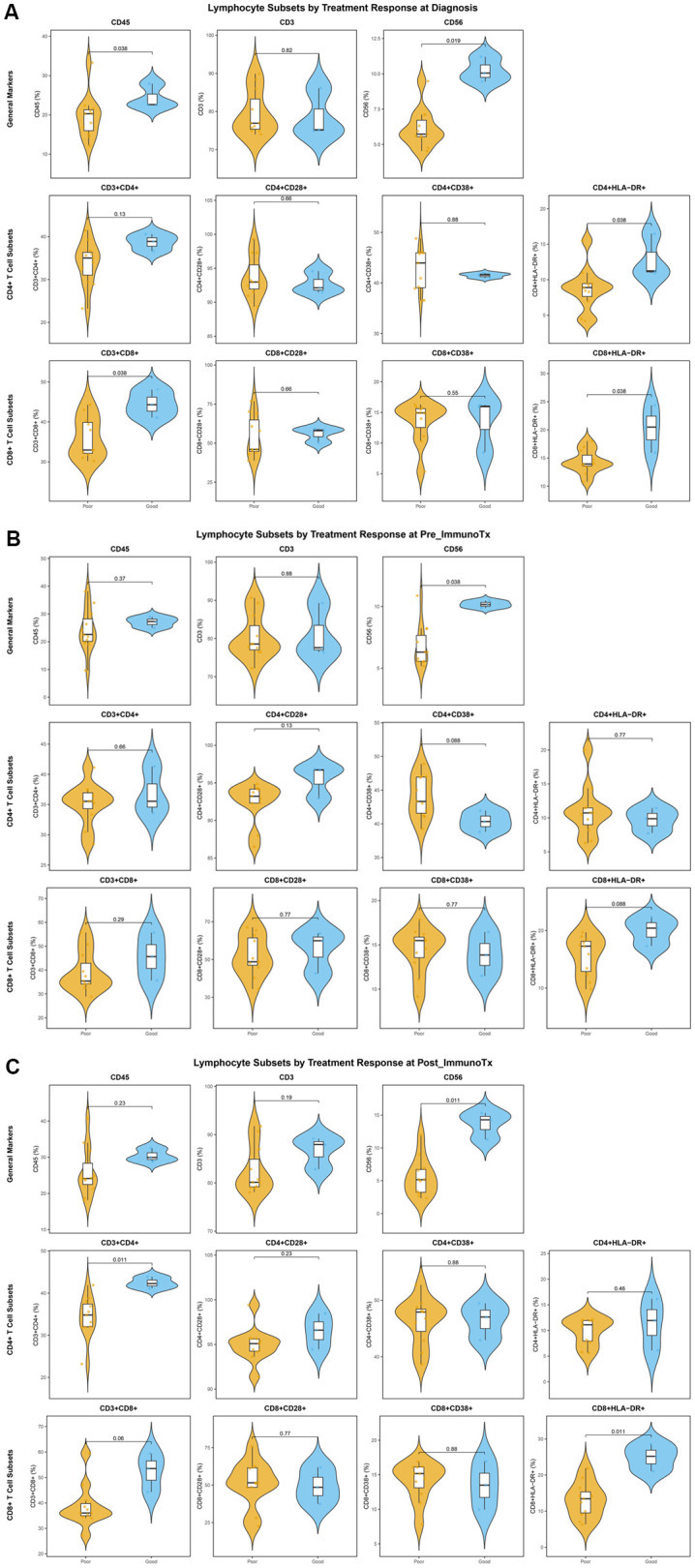
Violin plots illustrating the differences in peripheral blood lymphocyte subset proportions between DCR and PD groups in metastatic OSA patients. **(A)** Violin plots showing the distribution and differences of lymphocyte subset proportions at diagnosis between the DCR and PD groups; **(B)** Violin plots showing the distribution and differences of lymphocyte subset proportions before ICIs treatment between the DCR and PD groups; **(C)** Violin plots showing the distribution and differences of lymphocyte subset proportions after ICIs treatment between the DCR and PD groups.

We compared the levels of various lymphocyte subsets at three time points in patients in the DCR group and the PD group. The results showed that at diagnosis, the proportions of CD45^+^ cells (p = 0.038), CD3^-^CD56^+^ cells (p = 0.019), CD4^+^HLA-DR^+^ cells (p = 0.038), CD3^+^CD8^+^ cells (p = 0.038), and CD8^+^HLA-DR^+^ cells (p = 0.038) were significantly higher in the DCR group than in the PD group. However, before the first ICIs treatment, only the proportion of CD3^-^CD56^+^ cells was significantly higher in the DCR group than in the PD group (p = 0.038). Finally, after immunotherapy, the proportions of CD3^-^CD56^+^ cells (p = 0.011), CD3^+^CD4^+^ cells (p = 0.011), and CD8^+^HLA-DR^+^ cells (p = 0.011) were significantly higher in the DCR group than in the PD group (**Figures 3A–C**).

### 3.4 Dynamic trends of lymphocyte subsets during treatment

To further analyze the dynamic trends of lymphocyte subsets during ICIs treatment, a LMM was applied. The results revealed a significant difference in the trend of CD3^-^CD56^+^ cell proportion changes over time between different treatment response groups (DCR vs. PD) (Time Point × Response P < 0.001). Specifically, the proportion of CD3^-^CD56^+^ cells in the DCR group showed a significant increase after ICIs treatment, whereas in the PD group, the proportion either showed no significant change or a slight decrease.

**Figure 4:**
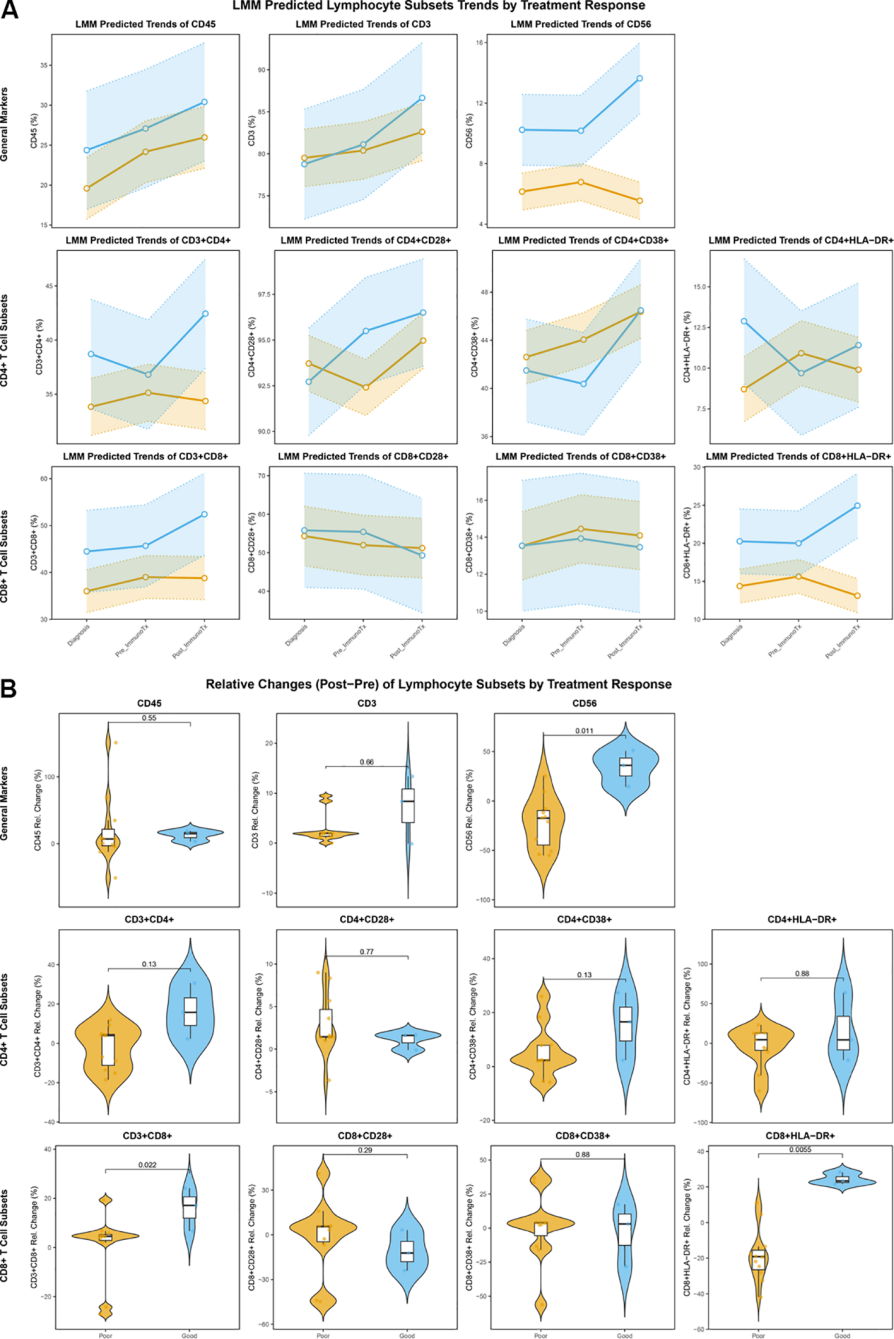
Dynamic trends and relative changes of peripheral blood lymphocyte subsets during ICIs treatment. **(A)** LMM analysis of lymphocyte subset proportions across three time points in DCR and PD groups. This line plot illustrates the dynamic changes of various lymphocyte subsets at diagnosis, before ICIs treatment, and after ICIs treatment. The yellow lines represent the PD group, and the blue lines represent the DCR group, highlighting distinct trajectories of lymphocyte subsets between the two response groups. Significant interaction effects (Time Point × Response) indicate differential dynamic changes over time; **(B)** Violin plots showing the differences in relative changes of lymphocyte subset proportions between the DCR and PD groups after ICIs treatment compared to before ICIs treatment. Each violin plot displays the distribution of the calculated relative change for a specific lymphocyte subset. The relative change was calculated as (value after ICIs - value before ICIs)/value before ICIs × 100%. These plots highlight which lymphocyte subsets show differential magnitudes of change between patients achieving clinical benefit versus those with progressive disease.

Similarly, the trend of CD8^+^HLA-DR^+^ cell proportion changes over time also differed significantly between the DCR and PD groups (Time Point × Response P = 0.003). The proportion of CD8^+^HLA-DR^+^ cells in the DCR group was significantly upregulated after ICIs treatment, suggesting an increase in activated T cells, while no obvious change was observed in the PD group. These results indicate that the dynamic patterns of CD3^-^CD56^+^ and CD8^+^HLA-DR^+^ cells are closely associated with clinical benefit from ICIs (**Figure 4A**).

### 3.5 Predictive ability of relative changes in lymphocyte subsets

We further calculated the relative change values of each lymphocyte subset after ICIs treatment compared to before ICIs treatment.

Regarding the prediction of treatment response, CD3^-^CD56^+^ cells (p = 0.011), CD3^+^CD8^+^ cells (p = 0.022), and CD8^+^HLA-DR^+^ cells (p = 0.005) were significantly increased in the DCR group after immunotherapy (**Figure 4B**). The analysis of absolute changes in PBLS before and after ICIs therapy also yielded similar results (**Supplementary Figure 1**).

**Figure 5:**
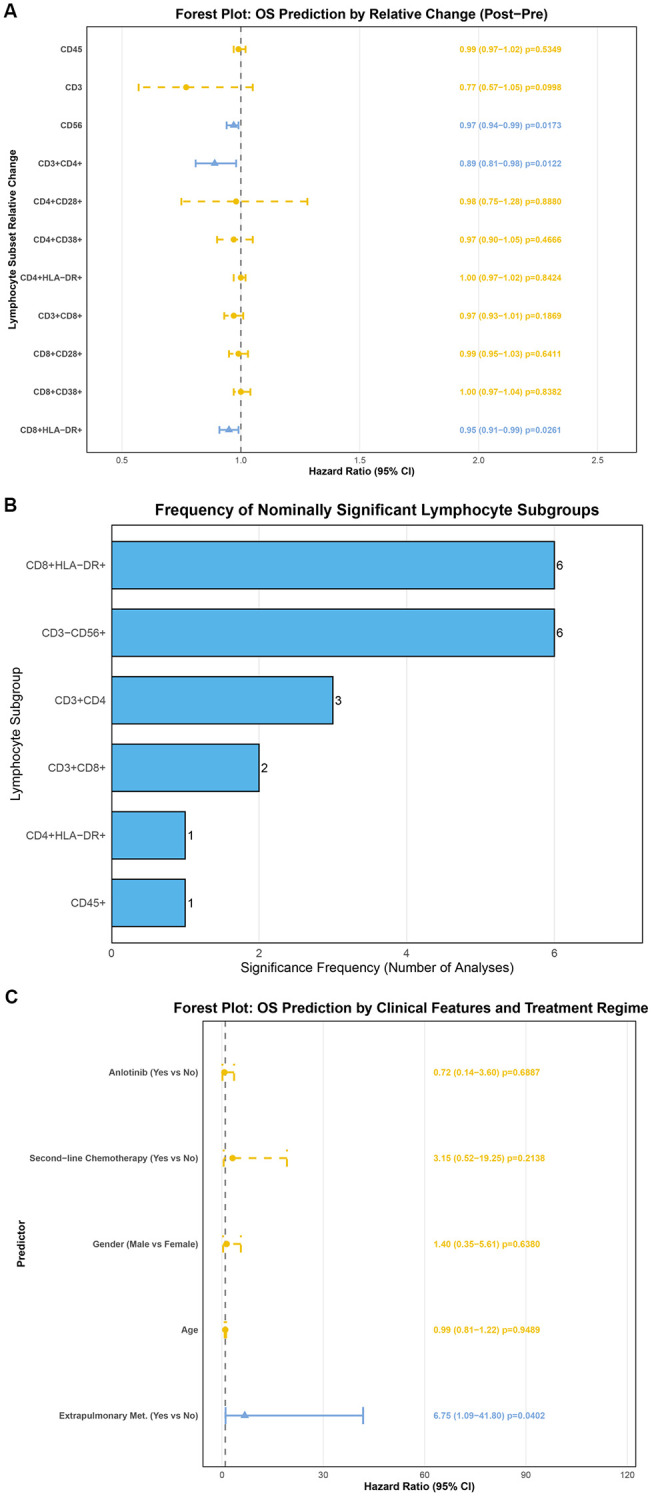
Predictive value of relative changes in lymphocyte subsets, frequency of significance, and clinical characteristics for overall survival. **(A)** Forest plot showing the univariate Cox regression analysis of relative changes in lymphocyte subset proportions after ICIs treatment (compared to before ICIs) and patient OS; **(B)** Bar plot illustrating the frequency with which each lymphocyte subset achieved statistical significance (P < 0.05) across multiple analytical dimensions. This plot summarizes the consistency of association signals for different lymphocyte subsets with patient prognosis and treatment response, as identified in various analyses throughout the study. The height of each bar corresponds to the number of times a particular subset showed statistical significance; **(C)** Forest plot showing the univariate Cox regression analysis of baseline clinical characteristics and patient OS.

Through Cox regression analysis, changes in certain lymphocyte subsets before and after immunotherapy were found to be associated with patient OS. Specifically, an increase in CD3^-^CD56^+^ cells (HR: 0.97 (0.94-0.99), p=0.017), CD3^+^CD4^+^ cells (HR: 0.89 (0.81-0.98), p=0.012), and CD8^+^HLA-DR^+^ cells (HR: 0.95 (0.91-0.99), p=0.026) were identified as protective factors for metastatic OSA patients undergoing ICIs treatment (**Figure 5A**).

Due to the limited sample size of this study, to preliminarily screen for lymphocyte subsets associated with ICIs efficacy and prognosis, the frequency with which each subset achieved statistical significance (P < 0.05) in the aforementioned analyses was tallied. The results are shown in **Figure 5B**. Among these, CD3^-^CD56^+^ cells and CD8^+^HLA-DR^+^ cells showed statistical significance in as many as 6 analyses, indicating a consistent association signal with patient prognosis and treatment response.

### 3.6 Relationship between clinical characteristics and ICIs efficacy and prognosis

Finally, we analyzed the relationship between various clinical characteristics and patient OS and treatment response. Univariate analysis results showed that only extrapulmonary metastasis was a risk factor for OSA patients (HR: 6.75 (1.09-41.80), p=0.040). Other clinical characteristics, including age, sex, use of anlotinib, and receipt of second-line chemotherapy, were not significantly associated with patient OS (**Figure 5C**).

## 4 Discussion

This study provides the first systematic analysis of dynamic changes in peripheral blood lymphocyte subsets in patients with metastatic OSA undergoing ICIs therapy. Our core findings indicate a potential association between elevated proportions of peripheral blood CD3^-^CD56^+^ and CD8^+^HLA-DR^+^ cells after ICIs treatment and longer OS and better treatment response. Supporting these core findings, statistical analysis of the frequency of significant findings across multiple analytical dimensions revealed a significant correlation between CD3^-^CD56^+^ and CD8^+^HLA-DR^+^ cells and patient OS at multiple time points. Furthermore, significant differences were repeatedly observed between the DCR group and the PD group, thereby highlighting their potential as biomarkers. In-depth linear mixed-model analysis further confirmed that the dynamic trends of CD3^-^CD56^+^ and CD8^+^HLA-DR^+^ cells in patients achieving clinical benefit differed significantly from those in patients experiencing disease progression. Regarding clinical features, extrapulmonary metastasis was identified as an independent risk factor for poor patient OS and unfavorable treatment response. Conversely, anlotinib use, second-line chemotherapy, age, and sex did not show significant associations with ICIs efficacy and prognosis.

The findings of this study provide crucial insights into the immune response mechanisms of metastatic OSA patients undergoing ICIs treatment. CD3^-^CD56^+^ cells primarily represent NK cells, which are key components of the innate immune system and directly recognize and kill tumor cells without prior sensitization (27, 28). In various tumor types, NK cell activity and count have been confirmed to be closely associated with patient prognosis and the efficacy of immunotherapy. For instance, Qu et al. observed that highly dynamic changes in NK cell percentage served as an independent prognostic predictor in hepatocellular carcinoma patients receiving ICIs treatment (29). Similarly, a study by Yi et al. revealed that NK cells were independent predictors of immunotherapy efficacy and progression-free survival in non-small cell lung cancer patients (30). Consistent with these existing findings, our study found that an increased proportion of CD3^-^CD56^+^ cells after ICIs treatment was significantly associated with better OS and treatment response. This suggests that the observed increase in NK cell proportions may be associated with the therapeutic effect of ICIs, potentially reflecting a more active role of NK cells in anti-tumor immunity. Although OSA is often considered a ‘cold tumor,’ research by Shrestha et al. demonstrated that NK cells play a critical role in overcoming its immunosuppressive microenvironment, further supporting the potential importance of NK cells in the response to ICIs treatment for OSA in this study (31).

CD8^+^HLA-DR^+^ cells are generally considered activated cytotoxic T lymphocytes (CTLs), and the high expression of HLA-DR molecules is an important marker of T cell activation and enhanced function (32, 33). CTLs can directly recognize and kill tumor cells and are the main executors of anti-tumor immune effector function (34). Ample evidence indicates a strong association between CD8^+^HLA-DR^+^ cells and therapeutic responses across various tumor types. For example, a study by Chi et al. showed that CD8^+^HLA-DR^+^ T cells were associated with better prognosis in non-small cell lung cancer and melanoma cohorts treated with anti-PD-1 therapy (35). Osuna-Gómez et al. further found that CD8^+^HLA-DR^+^ T cells were significantly increased in responders to neoadjuvant chemotherapy for breast cancer (33). Consistent with these findings, this study observed that the increased proportion of CD8^+^HLA-DR^+^ cells after ICIs treatment was associated with clinical benefit and longer OS, strongly suggesting a potential association between ICIs treatment and the expansion of cytotoxic T cell responses in patients, thereby mediating effective clearance of tumors (36, 37).

The treatment of metastatic OSA remains one of the most formidable challenges in clinical oncology. Given the transformative efficacy of ICIs in common solid tumors such as melanoma, lung cancer, and cervical cancer, researchers have actively introduced them into the clinical exploration of OSA (5, 6). However, multiple clinical trials have demonstrated that the ORR of ICI monotherapy in unselected OSA patients is extremely limited. For instance, the OSA cohort in the study by Davis et al. showed no objective responses; in the SARC028 trial (NCT02301039), only one out of 22 patients exhibited an objective response; another phase II trial for recurrent OSA (NCT03013127) also confirmed that, despite good tolerability, pembrolizumab failed to provide significant clinical benefit (11–13). Given the limitations of monotherapy, researchers have further explored combination strategies: Le Cesne et al. investigated the combination of pembrolizumab and cyclophosphamide, which resulted in only one partial response; similarly, the study by Palmerini et al. on nivolumab combined with sunitinib showed a trend toward superiority over monotherapy in certain subtypes, yet the overall ORR remained far below that observed in common solid tumors (10, 14). These results underscore the urgent need to develop predictive biomarkers to identify populations that may potentially benefit.

The search for precise predictive biomarkers in the field of OSA has been arduous. For example, PD-L1 expression and tumor mutational burden, which have proven effective in common solid tumors like non-small cell lung cancer, face severe applicability challenges in OSA (38, 39). Regarding PD-L1, although a Tumor Proportion Score (TPS) of ≥50% is often used as the decision threshold for first-line immunotherapy in lung cancer, PD-L1 expression is generally extremely low in OSA (40). Multiple studies have shown that the TPS in OSA patients is often below 10%, with some studies even defining a TPS ≥5% as positive (15, 41). More critically, clinical evidence indicates that the anti-tumor activity of pembrolizumab in OSA does not depend on PD-L1 expression status, which directly undermines its clinical utility as a predictive indicator (12, 42). Meanwhile, the mean TMB in OSA is only 0.79–1.96 mut/Mb, far below the threshold associated with immunotherapy benefit, further limiting the potential of TMB in clinical decision-making for OSA (43, 44). Although the emerging 7-gene signature has shown encouraging predictive potential in sarcoma cohorts, its clinical translation remains hindered by technical bottlenecks, specifically the lack of standardized optimal normalization methods across sequencing platforms (19). Consequently, recent investigations have explored standardized measurements derived from routine blood tests as alternative approaches to assess ICIs efficacy. For example, Yoo et al. developed the SCORPIO system, which, based on routine blood tests and clinical data, demonstrated superior performance in predicting ICIs efficacy compared to PD-L1 immunostaining (22).

Traditional biomarker studies often prioritize single, pre-treatment measurements, overlooking the significance of dynamic monitoring (45). However, tumor immunotherapy itself is a highly dynamic process, and the patient’s immune status undergoes significant and continuous changes during treatment (24, 46, 47). This study innovatively undertook the systematic collection of peripheral blood lymphocyte subset proportions from OSA patients at multiple key time points during ICIs treatment. An in-depth analysis of the dynamic changes in these lymphocyte subsets was then performed using linear mixed models, and relative change values pre- and post-treatment were calculated. Our findings suggest that, in single-time-point analyses, post-immunotherapy markers may exhibit greater predictive value than pre-treatment or diagnostic markers. Furthermore, through analysis of the dynamic changes in lymphocyte subsets before and after immunotherapy, these dynamic indicators were found to effectively predict the response to immune checkpoint inhibitor therapy and patient prognosis. This underscores the importance of dynamic monitoring of peripheral blood immune cell subsets, as it provides a more accurate and comprehensive reflection of the immune system’s response to ICIs treatment, ultimately facilitating more informed and timely clinical decision-making (48, 49).

Currently, research on the efficacy of ICIs in metastatic OSA and its predictive biomarkers is relatively scarce. While existing small-scale clinical trials and retrospective studies indicate that the overall efficacy of ICIs in sarcoma is generally lower compared to other tumor types like melanoma or lung cancer, a subset of patients still experiences durable clinical benefits (50, 51). Our findings align closely with research in other tumor types (e.g., melanoma, non-small cell lung cancer) demonstrating a correlation between immune cell subsets and ICIs efficacy, specifically, that an increase in activated T cells and NK cells typically predicts improved clinical outcomes (52, 53).

This study found that non-pulmonary metastasis is a risk factor for poor OS and suboptimal treatment response in patients with metastatic OSA. This is consistent with existing clinical observations and research results (54). Non-pulmonary metastasis (e.g., bone, liver, or brain metastasis) typically indicates a higher tumor burden, a more advanced disease stage, and a more aggressive tumor phenotype (55). These metastatic lesions may have more complex immunosuppressive microenvironments, such as higher infiltration of myeloid-derived suppressor cells (MDSCs) and tumor-associated macrophages (TAMs), and lower T cell infiltration, thereby generating resistance to ICIs therapy (56, 57). In addition, tumor cells in different metastatic sites may exhibit distinct genomic characteristics and immunogenicity, leading to varying sensitivities to immunotherapy (58, 59). Therefore, OSA patients with non-pulmonary metastasis may require more aggressive or combined treatment strategies to mitigate the challenges associated with their poor prognosis.

The potential impact of prior and concomitant treatments on peripheral blood lymphocyte subsets represents a complex variable in our study. Chemotherapy is frequently associated with alterations in the immune landscape; for instance, studies have reported that regimens such as R-CHOP may correlate with reduced counts of CD3^+^, CD4^+^, CD8^+^ T cells, and NK cells (60). Furthermore, the effect of chemotherapy on lymphocyte subsets appears to be context-dependent, as evidenced by Hong et al., who observed varying impacts on NK and T cell proportions depending on the treatment cycle and regimen (61). These findings suggest that chemotherapy may modulate the immune profile, potentially confounding the interpretation of lymphocyte dynamics in our cohort. Similarly, tyrosine kinase inhibitors (TKIs) like anlotinib, while generally exhibiting less systemic cytotoxicity than traditional chemotherapy, may also modulate the immune microenvironment (62). Literature has observed that TKI therapy can correlate with shifts in immune cell populations, such as increases in NK cell counts or CD8^+^ CTL proportions in various malignancies (63). While these observations provide a biological rationale for the immune changes seen in our study, they also highlight that prior or concomitant TKI use could act as a confounding factor. Due to the limited sample size (n=14), we were unable to perform multivariate adjustment to isolate the independent effects of these prior treatments from the immunotherapy response. Consequently, while our findings suggest a potential association between lymphocyte dynamics and ICIs efficacy, we cannot definitively exclude the possibility that prior chemotherapy or anlotinib use contributed to the observed immune profiles.

While this study yields meaningful findings, its limitations warrant careful consideration. First, the limited sample size (n=14) and the retrospective nature of the study constitute the most significant limitations. These factors inherently introduce risks of selection bias and variable data integrity, while also resulting in insufficient statistical power, which may increase the risk of spurious findings and hinder the detection of subtle differences. Consequently, these findings necessitate rigorous validation in a larger, prospective cohort. Second, our analysis was restricted to routine clinical immunophenotyping panels, which are standardized for clinical practice and primarily focus on surface antigens. This methodology precluded the assessment of intracellular functional markers, such as Granzyme B, which are known to be critical for cytotoxic activity. Furthermore, while we observed numerical trends in several other lymphocyte subsets, these did not reach statistical significance. This is likely attributable to a combination of the limited statistical power in our small cohort and the biological nature of these markers. For instance, HLA-DR is a classic marker of late-stage T-cell activation, potentially making it more dynamically responsive to the sustained immune activation induced by ICIs compared to markers like CD28, which are constitutively expressed on most T cells and may exhibit less dynamic fluctuation in this clinical setting. Given the evolving understanding of the tumor microenvironment, future studies integrating more comprehensive biomarker panels—including both surface and intracellular markers—are essential to fully elucidate the immune landscape of metastatic OSA.

Nevertheless, this study retains considerable value. The first systematic analysis of dynamic changes in peripheral blood lymphocyte subsets at three critical time points—diagnosis, pre- ICIs treatment, and post-ICIs treatment—in patients with metastatic OSA was conducted. This offers a novel perspective on the immune response of OSA to ICIs.

In light of these limitations, future research should prioritize the following areas. First, larger-scale, multi-center, prospective clinical trials are essential to validate the effectiveness and reliability of the identified lymphocyte subsets as predictive biomarkers. Second, future investigations should integrate tumor histological analysis (e.g., density and phenotype of tumor-infiltrating lymphocytes, PD-L1 expression, TMB) with comprehensive omics data (e.g., genomics, transcriptomics, metabolomics) to elucidate the mechanisms underlying OSA response to ICIs from multiple perspectives and identify more robust biomarker panels. Finally, the development of advanced biomarker-based assays for peripheral blood lymphocyte subset analysis is crucial for a more precise characterization of lymphocyte functional subsets.

## 5 Conclusion

In conclusion, this study offers pioneering insights into the dynamic interplay between peripheral blood lymphocyte subsets and the efficacy of immune checkpoint inhibitors in metastatic OSA. We systematically demonstrate that the absolute proportions and, more critically, the dynamic increases of CD3^−^CD56^+^ NK cells and CD8^+^HLA-DR^+^ activated cytotoxic T cells after ICIs treatment are consistently associated with improved overall survival and favorable treatment responses. These findings underscore the importance of dynamic immune monitoring, moving beyond single-time-point assessments, to better understand and predict patient outcomes in a highly challenging disease context. While acknowledging the limitations of a small sample size, this research provides a robust preliminary foundation for identifying accessible and clinically relevant biomarkers that could facilitate personalized risk stratification and treatment decision-making for metastatic OSA patients receiving ICIs. Future large-scale, multi-center prospective studies are warranted to validate these findings and further integrate multi-omics data for a comprehensive understanding of OSA immune responses.

## Data Availability

The raw data supporting the conclusions of this article will be made available by the authors, without undue reservation.
