# Supplementary figures and images for: Dynamic changes in peripheral blood lymphocyte subsets predict the efficacy and prognosis of immune checkpoint inhibitors in metastatic osteosarcoma
Source: Front Immunol. 2026 May 13;17:1766639. doi: 10.3389/fimmu.2026.1766639 (PMC13212053; doi:10.3389/fimmu.2026.1766639)

**CD45+**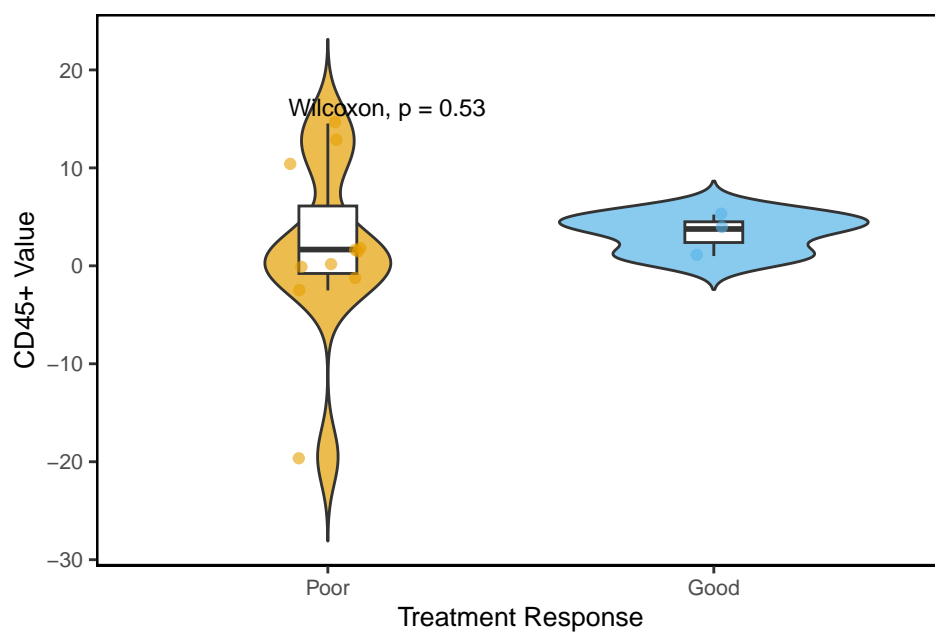**CD3+**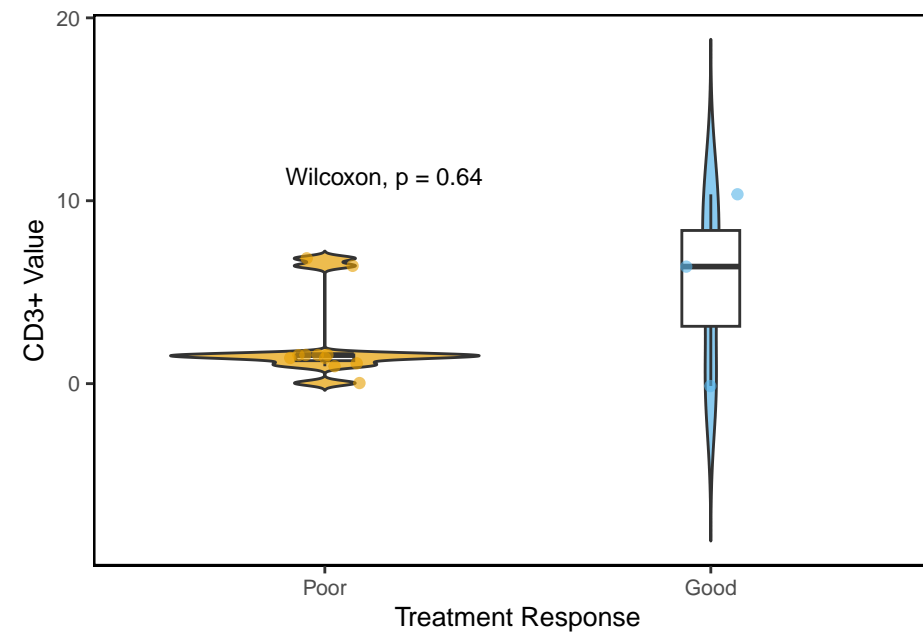**CD3-CD56+**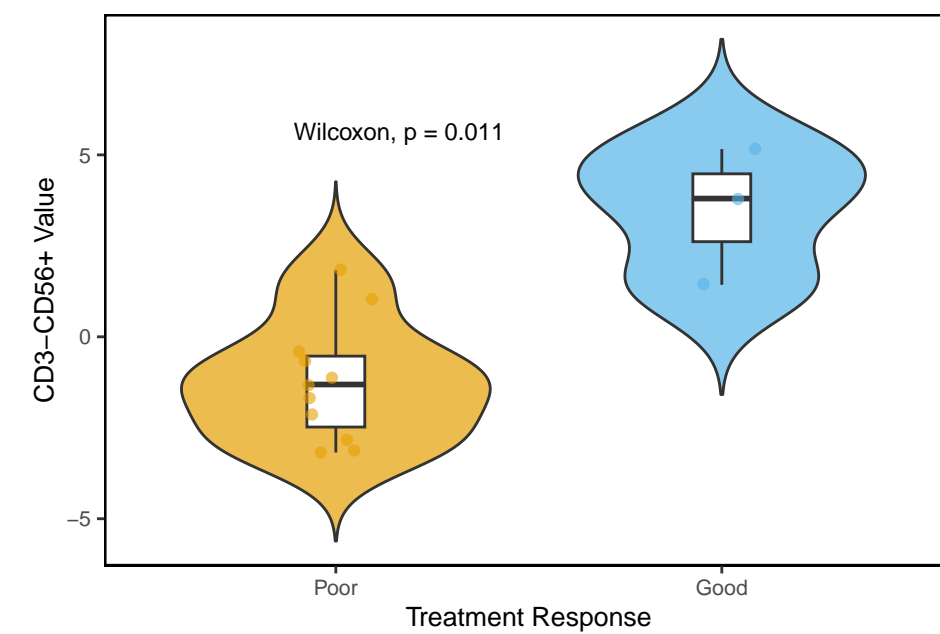**CD3+CD4+**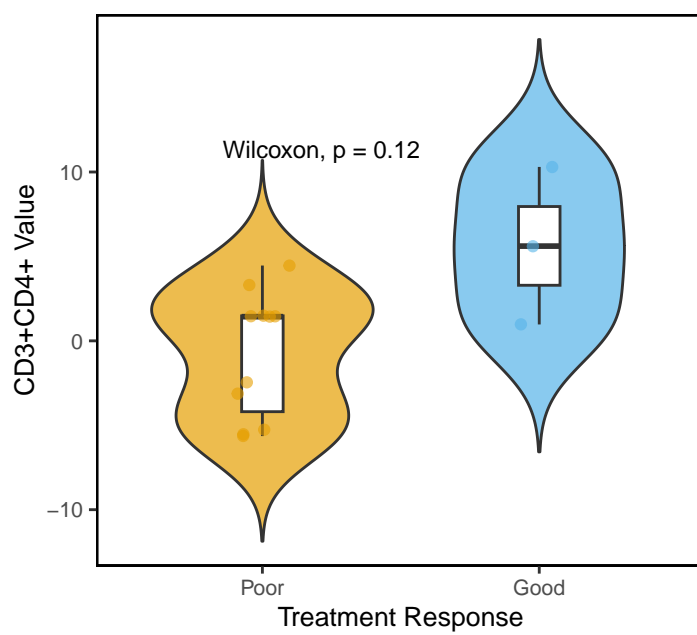**CD4+CD28+**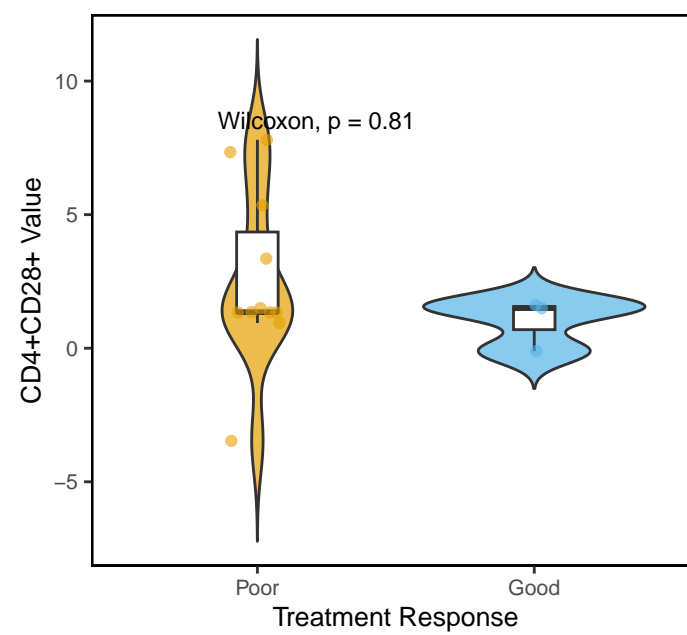**CD4+CD38+**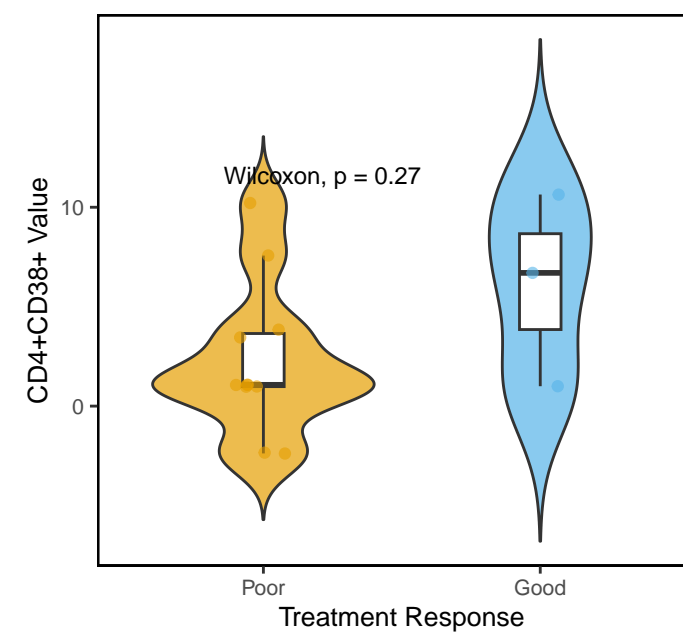**CD4+HLA-DR+**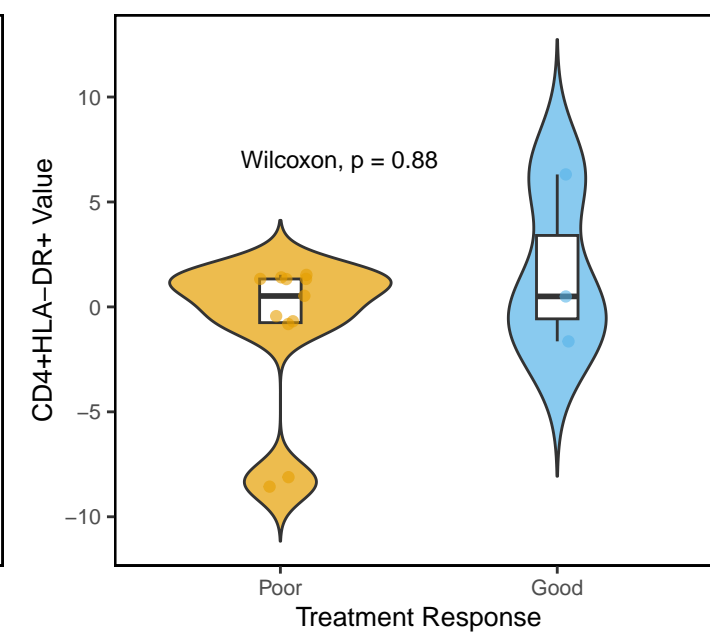**CD3+CD8+**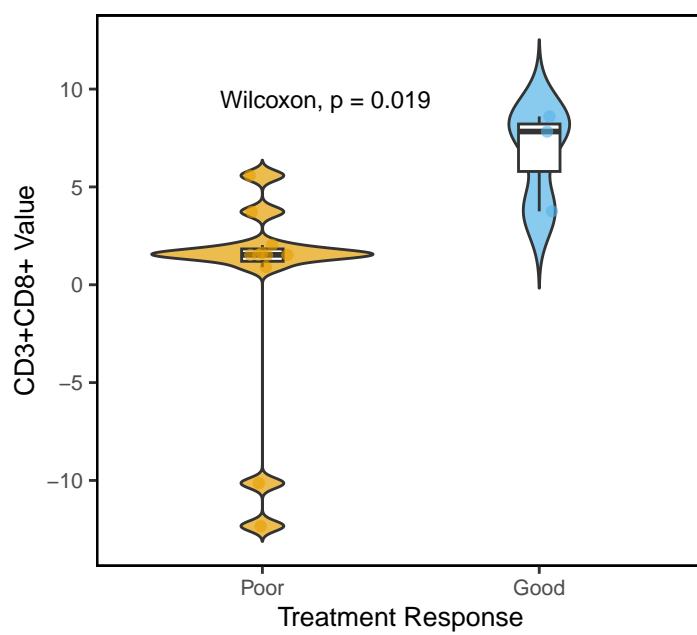**CD8+CD28+**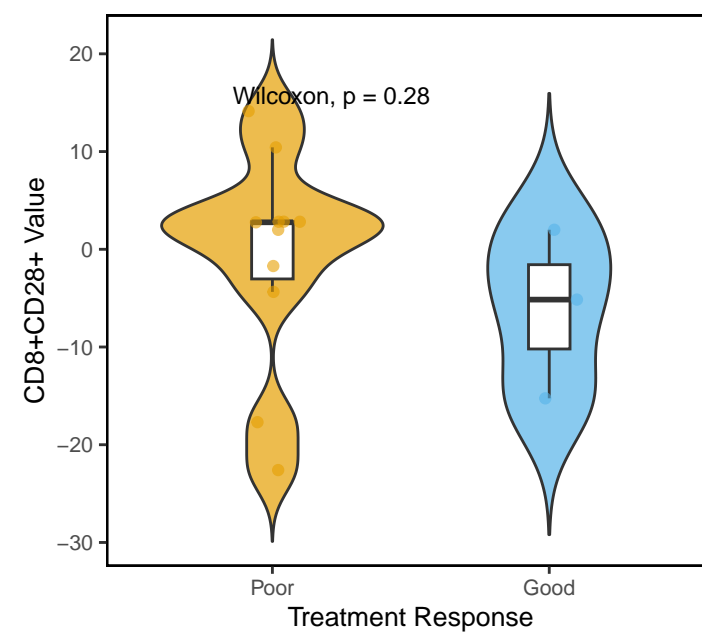**CD8+CD38+**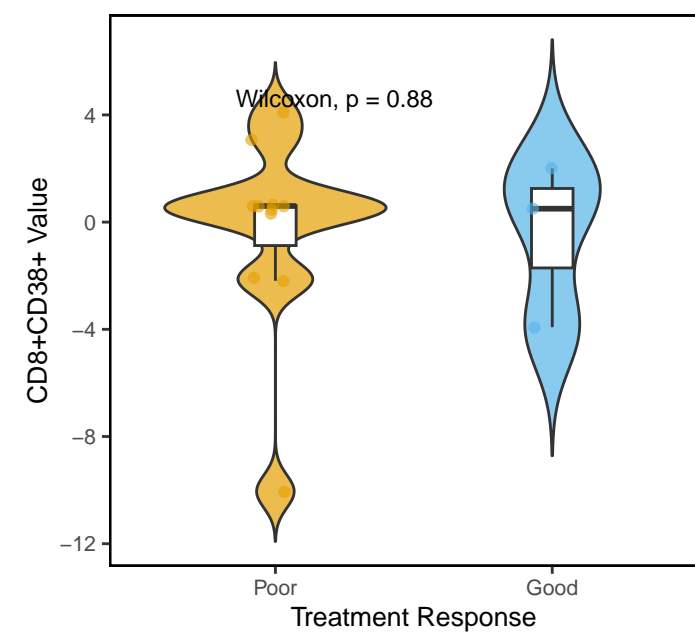**CD8+HLA-DR+**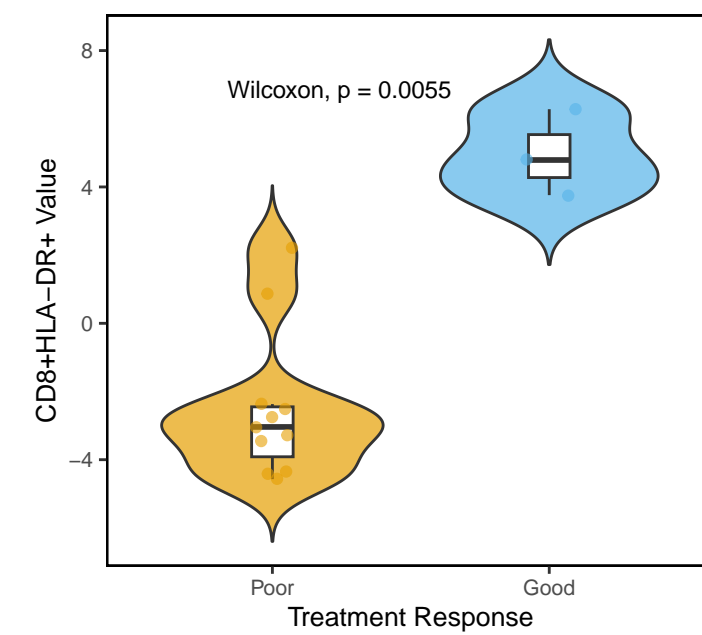

Supplement: Supplementary file 1 [file Image1.pdf]
