# Supplementary material for: Dynamic changes in peripheral blood lymphocyte subsets predict the efficacy and prognosis of immune checkpoint inhibitors in metastatic osteosarcoma
Source: Front Immunol. 2026 May 13;17:1766639. doi: 10.3389/fimmu.2026.1766639 (PMC13212053; doi:10.3389/fimmu.2026.1766639)
Supplement: Supplementary file 2 [file Table1.docx]

**Flow cytometry methodology**

**The antibodies used were as follows:**

FITC anti-human CD45 Antibody Biolegend

PE/Cyanine7 anti-human CD3 Antibody Biolegend

PerCP/Cyanine5.5 anti-human CD4 Antibody Biolegend

APC anti-human CD8 Antibody Biolegend

PE anti-human CD56 (NCAM) Antibody Biolegend

APC/Cyanine7 anti-human CD28 Antibody Biolegend

Brilliant Violet 421™ anti-human CD38 Antibody Biolegend

Brilliant Violet 510™ anti-human HLA-DR Antibody Biolegend

**the gating strategy was as follows:**

**Lymphocyte Gating:** Lymphocytes were identified based on their characteristic low forward scatter (FSC) and low side scatter (SSC) properties.

**Singlet Gating:** Doublets were excluded using FSC-H vs. FSC-A plots.

**Leukocyte Identification:** CD45^+^ cells were gated to distinguish leukocytes from debris.

**Major Subsets:** T cells were identified as CD3^+^ cells, and NK cells were identified as CD3^-^CD56^+^ cells.

**T-cell Subsets:** Within the CD3^+^ population, CD4^+^ and CD8^+^ T cells were further gated.

**Activation Status:** Activation markers (HLA-DR, CD38, CD28) were analyzed within the CD4^+^ and CD8^+^ T-cell populations (e.g.,CD4^+^HLA-DR^+^, CD8^+^HLA-DR^+^).
